# The Influence of Countermovement Jump Protocol on Reactive Strength Index Modified and Flight Time: Contraction Time in Collegiate Basketball Players

**DOI:** 10.3390/sports7020037

**Published:** 2019-02-12

**Authors:** Aaron Heishman, Brady Brown, Bryce Daub, Ryan Miller, Eduardo Freitas, Michael Bemben

**Affiliations:** 1 Department of Health and Exercise Science, University of Oklahoma, Norman, OK 73019, USA; brownbrady3@ou.edu (B.B.); ryanmiller1@ou.edu (R.M.); eduardofreitas@ou.edu (E.F.); mgbemben@ou.edu (M.B.); 2 Department of Athletics, Basketball Strength and Performance, University of Oklahoma, Norman, OK 73019, USA; bdaub34@gmail.com

**Keywords:** athlete monitoring, athlete performance, collegiate basketball, fatigue monitoring, countermovement jump, CMJ arm swing, CMJ without arm swing

## Abstract

The purpose of the present investigation was to evaluate differences in Reactive Strength Index Modified (RSI_Mod_) and Flight Time to Contraction Time Ratio (FT:CT) during the countermovement jump (CMJ) performed without the arm swing (CMJNAS) compared to the CMJ with the arm swing (CMJAS), while exploring the relationship within each variable between jump protocols. A secondary purpose sought to explore the relationship between RSI_Mod_ and FT:CT during both jump protocols. Twenty-two collegiate basketball players performed both three CMJNAS and three CMJAS on a force plate, during two separate testing sessions. RSI_Mod_ was calculated by the flight-time (RSI_Mod_^FT^) and impulse-momentum methods (RSI_Mod_^IMP^). CMJ variables were significantly greater during the CMJAS compared to CMJNAS (*p* < 0.001). There were large to very large correlations within each variable between the CMJAS and CMJNAS. There were significant positive correlations among RSI_Mod_^FT^, RSI_Mod_^IMP^, and FT:CT during both the CMJAS (r ≥ 0.864, *p* < 0.001) and CMJNAS (r ≥ 0.960, *p* < 0.001). These findings identify an increase in RSI_Mod_ or FT:CT during the CMJAS, that may provide independent information from the CMJNAS. In addition, either RSI_Mod_ or FT:CT may be utilized to monitor changes in performance, but simultaneous inclusion may be unnecessary.

## 1. Introduction

The countermovement jump (CMJ) is routinely used by both practitioners and researchers to monitor acute and long-term changes in athlete performance. The CMJ offers a non-invasive assessment that can be performed in a time efficient manner, making it an attractive field measure to evaluate neuromuscular performance [1]. In addition, the CMJ involves the dynamic muscle action known as the stretch-shortening cycle (SSC), which is a key component in many sporting events [2]. More specifically, the CMJ has been associated with slow-SSC (>250 ms in duration) performance, which has been related to sprint acceleration, where ground contact time is longer [3,4]. This makes the CMJ assessment a fundamental tool in appraising key performance indices among basketball athletes, which require frequent acceleration and deceleration.

Two protocols are regularly employed when performing the CMJ. One protocol is performed without an arm swing (CMJ NAS), which requires the athlete to maintain hand placement on their hips or grasping a practically-weightless implement (e.g., polyvinyl chloride pipe or wood dowel) positioned on their shoulders during the CMJ [5,6,7,8,9]. The CMJ NAS method isolates lower extremity force production by mitigating the influence of the arm swing. Alternatively, the second CMJ method incorporates the use of the arm swing (CMJ AS), with previous literature advocating the inclusion of the arm swing as it may reflect a greater degree of sport specificity and familiarity among skilled jumpers [10,11,12,13]. Previous literature has supported the intra- and inter-session reliability of the majority of CMJ variables during both CMJ protocols, especially in skilled jumpers [5,7,10,14,15]. However, the arm swing appears to positively influence performance during the CMJ, such as increasing jump height and velocity at take-off, when compared to the CMJ NAS [16,17,18,19]. These noted increases in performance may allude to an athlete’s absolute maximal capacities, which could provide additionally pertinent information during an athlete’s needs analysis useful in directing a training program.

Reactive Strength Index Modified (RSI_Mod_) and the Flight Time to Contraction Time Ratio (FT:CT) are two common variables of interest during CMJ analysis. Adopted from the Reactive Strength Index during the drop-jump, RSI_Mod_ assesses an athlete’s ability to create maximal vertical impulse in a minimal amount of time during the CMJ, while being credited as a valid measure of lower extremity explosiveness as it includes factors of both force and speed [3,20,21]. RSI_Mod_ is calculated by dividing jump height (JH) by contraction time (CT) [16]. JH can be computed via the flight time method, where the time the athlete is in flight is measured to determine jump height, and then used to generate RSI_Mod_^FT^, or by quantifying JH from the impulse-momentum method, which required the use of force platforms and used to produce RSI_Mod_^IMP^ [20,22]. CT is defined as the duration (ms) from jump initiation (start of movement) to take-off [10]. Similarly, FT:CT compares the ratio of an outcome variable (FT) and a process variable (CT) in an effort to evaluate an athlete’s jumping strategy, ultimately alluding to neuromuscular readiness [20]. Previous literature has utilized both RSI_Mod_ and FT:CT to evaluate athlete performance and monitor neuromuscular functional status [23,24,25]. In fact, RSI_Mod_ and FT:CT are oftentimes utilized over traditional gross output measures (i.e., force, power, jump height, etc.), as they may provide more relevant information reflecting changes in movement strategy in an attempt to meet the gross output desired [5]. However, the similarities in computation between the two metrics may make their simultaneous inclusion in analysis redundant for athlete monitoring and performance testing. Nevertheless, previous literature has yet to compare performance differences in RSI_Mod_ and FT:CT between CMJ protocols, nor the relationship in performance within each variable between the CMJ NAS the CMJ AS. Understanding the relationship within each variable between protocols may aid coaches in determining which CMJ protocol to use for athlete assessment, while also providing context for practitioners when comparing an athlete’s performance profile characteristics with respect to normative data [26].

Recent work by McMohan et al. [20] demonstrated a strong correlation between the variables of FT:CT and RSI_Mod_ when performing the CMJ NAS in a cohort of sport science graduate students. However, the maintenance of this relationship during the CMJ AS, where differences in mechanical events of the CMJ are notably different, and among a cohort of skilled jumpers remains unknown. In addition, previous literature has yet to explore differences in RSI_Mod_^FT^, RSI_Mod_^IMP^, and FT:CT between the CMJ NAS and CMJ AS protocols. Therefore, the primary purpose of the present investigation was to evaluate differences in RSI_Mod_^FT^, RSI_Mod_^IMP^, and FT:CT during the CMJ NAS compared to the CMJ AS, while additionally exploring the relationship within each variable between jump protocols. In addition, a secondary purpose sought to explore the relationship among RSI_Mod_^FT^, RSI_Mod_^IMP^, and FT:CT during both the CMJ AS and the CMJ NAS. It was hypothesized RSI_Mod_^FT^, RSI_Mod_^IMP^, and FT:CT would be significantly greater during the CMJ AS, while exhibiting moderate to large correlations between CMJ protocols. Secondarily, it was hypothesized RSI_Mod_^FT^, RSI_Mod_^IMP^, and FT:CT would demonstrate strong positive correlations during both CMJ NAS and CMJ AS, while more specifically, the relationship between RSI_Mod_^FT^ and RSI_Mod_^IMP^ would produce enhanced limits of agreement during the CMJ NAS compared to the CMJ AS.

## 2. Materials and Methods

### 2.1. Subjects

A convenience sample of twenty-two (Men: n = 14, age = 19.7 ± 1.0 years, height = 1.98 ± 0.71 m, body mass = 94.7 ± 6.2 kg; Women: n = 8, age = 20 ± 1.6 years, height = 1.80 ± 0.65 m, body mass = 78.2 ± 8.3 kg) NCAA Division 1 collegiate basketball players were included in this study. All subjects were active squad members of the University of Oklahoma’s Men’s and Women’s Basketball teams. This research was approved by the Institutional Review Board of the University of Oklahoma and all subjects provided written, informed consent before participating in the study.

### 2.2. Procedures

Data were collected using a randomized cross-over within subject study design. The dependent variables of interests were RSI_Mod_^FT^, RSI_Mod_^IMP^, and FT:CT and have been defined previously [10]. All testing took place within a 2-week time frame during the off-season training period. Subjects performed CMJ assessments during two different test sessions, with each test session, including three CMJ NAS and three CMJ AS, for a total of six CMJ during Test Session 1 and six CMJ during Test Session 2. The order of the jumps was randomly assigned, and subjects performed the jump type in the reciprocal order during the second session of testing. A minimum of 2 min of rest was allotted between jump trials. In addition, each subject performed both testing sessions in the afternoon within the same time of day between 13:00 and 14:00, as previous literature has identified the influence of time of day on jump performance [27]. In accordance with prior literature [7] and in an attempt to control the impact of training loads on testing outcomes, CMJ testing was performed within the same time-frame of the training week and training loads were strictly matched 72 h prior to both testing sessions, with sport-specific practice duration matched in the days prior to both trial sessions. Further, subjects were instructed to have no physical exertion prior to arriving on days of testing. In an effort to maintain ecological validity, subjects wore their standard practice gear, including shoes of their choosing, but each subject was required to wear the same pair of shoes during both testing sessions. Furthermore, no dietary restrictions were implemented, however athletes were instructed to maintain normal dietary intake, as outlined by the team’s sports nutritionist.

All testing was conducted at the basketball training facility prior to the start of strength training sessions. The same standardized warm-up was performed before each testing session, which included dynamic stretching and locomotion patterns (i.e., skipping, jogging and running), similar to previous literature [6,10]. Movement intensities gradually increased over the warmup duration to prepare participants for maximal performance during the jump testing. CMJs were performed on the ForceDecks FD4000 Dual Force Platforms hardware (ForceDecks, London, UK), with a sample rate of 1000 Hz. To limit the impact of instructions on the CMJ performance characteristics, consistent instructions were provided to all participants during each CMJ trial [28]. In addition, verbal encouragement was provided to support maximal effort during each jump attempt.

#### 2.2.1. Countermovement Jump with No Arm Swing (CMJ NAS)

The subject started in the tall standing position, with feet placed hip width to shoulder width apart and hands akimbo. The subject was then instructed to start with equal weight distribution on both force platforms. A visual representation of weight distribution was displayed on a monitor in front of the participant to provide synchronized and integrated feedback, allowing the participant to adjust their positioning for equal quantities of body weight to be distributed on each force platforms for the start of the jump. The subject then dropped into the countermovement position to a self-selected depth, followed by a maximal effort vertical jump, and landed in an athletic position on the force platforms. The subject reset to the starting position after each jump, and the procedure was completed for a total of three jumps. If at any point the subject removed their hands from their hips or exhibited excessive knee flexion once airborne, the jump was ruled invalid and repeated.

#### 2.2.2. Countermovement Jump with an Arm Swing (CMJ AS)

In the same manner as the CMJ NAS, subjects started in the tall standing position, with feet placed hip width to shoulder width apart, but with hands free for movement. The subject was then instructed to start with equal weight distribution on both force platforms. A visual representation of weight distribution was displayed on a monitor in front of the participant to provide synchronized and integrated feedback, allowing the subject to adjust their positioning for equal quantities of body weight to be distributed on each force platforms for the start of the jump. The subject then dropped into the countermovement position to a self-selected depth, incorporating an arm swing in their most natural, self-selected manner, followed by a maximal effort vertical jump and landing in an athletic position on the force platforms. The subject reset to the starting position after each jump, and the procedure was completed for a total of three jumps. If at any point the subject exhibited excessive knee flexion once airborne, the jump was ruled invalid and repeated.

### 2.3. Data Analysis

The commercially available ForceDecks software (ForceDecks, London, UK) was used to analyze all CMJs and generate the CMJ variables using conventional methods [29]. Before calculations are made, the ForceDecks Software combines the data from the two force transducers (sum of the left and right force data). The software uses a 20 N offset from the measured bodyweight obtained prior to the jump, to define the start of the movement. The end of eccentric and the start of concentric was defined as minimum displacement (absolute) which is equal to zero velocity, while take-off was defined as the timepoint at which total vertical force fell below the threshold of 20 N below bodyweight. Contraction time was calculated as the time interval between the onset of movement and take-off. Flight time was calculated as the time interval between take-off and touch down. RSI_Mod_^FT^ was calculated as jump height, determine by the conventional flight-time method (Jump Height^FT^ = 1/2 g(t/2)^2^, where g = gravitational acceleration and ft = flight time) divided by contraction time, therefore RSI_Mod_^FT^ = Jump Height^Flight Time^/Contraction Time [10,22,29,30]. RSI_Mod_^IMP^ was calculated as jump height, determined by the conventional impulse-momentum method (Jump Height^IMP^ = v^2^/2g, where v = velocity at take-off and g = gravitational acceleration), divided by contraction time, therefore RSI_Mod_^IMP^ = Jump Height^Impulse^/Contraction Time [22,29,31]. FT:CT was calculated as the flight time divided by contraction time.

### 2.4. Statistical Analysis

Statistical measures are reported as mean ± SD. Two-way (Condition (CMJ NAS vs. CMJ AS) X Time (Test Session 1 vs. Test Session 2)) repeated measures analyses of variance (ANOVA) with Bonferroni post hoc pairwise comparison used to determine significant condition and time main effects and significant condition by time interactions within the variables of RSI_Mod_^FT^, RSI_Mod_^IMP^ and FT:CT. Effects sizes (Cohen’s *d*) were calculated and interpreted as trivial (0–0.19), small (0.20–0.49), medium (0.50–0.79), and large (0.80 and greater) [32]. Pearson correlation coefficients were utilized to examine the relationship between the CMJ NAS and CMJ AS protocols within the RSI_Mod_^FT^, RSI_Mod_^IMP^ and FT:CT variables, during Test Session 1 and Test Session 2. In addition, Pearson correlation coefficients were used to examine relationships between the variables of RSI_Mod_^FT^, RSI_Mod_^IMP^ and FT:CT within each condition (CMJ NAS and CMJ AS), during Test Session 1 and Test Session 2. In accordance with previous literature, correlation coefficients were interpreted as trivial (0–0.09), small (0.10–0.29), moderate (0.30–0.49), large (0.50–0.69), very large (0.70–0.89), and almost perfect (0.90–1) [20,32,33]. Furthermore, a one-way repeated measures ANOVA was used to evaluate differences in RSI_Mod_^FT^ compared to RSI_Mod_^IMP^ during both the CMJ NAS and CMJ AS. A Bland-Altman Plot was utilized to assess the Limits of Agreement between RSI_Mod_^FT^ and RSI_Mod_^IMP^ during both the CMJ NAS and CMJ AS [34]. All statistical analyses were performed using SPSS software (Version 24; SPSS Inc., Chicago, IL, USA), with the alpha level set at *p* ≤ 0.05.

## 3. Results

The descriptive statistics are outlined in Table 1. Data normality was confirmed and is presented as the mean of the three jumps performed during each condition from Test Session 1 and Test Session 2. The inter- and intra-session reliability of the variables in the present study are reported elsewhere [10].

There was a significant condition (CMJ AS vs. CMJ NAS) effect, with RSI_Mod_^FT^ (*d* = 0.67; *p* < 0.001), RSI_Mod_^IMP^ (*d* = 0.66; *p* < 0.001), and FT:CT (*d* = 0.52; *p* < 0.001) all significantly greater during the CMJ AS compared to the CMJ NAS. However, there were no significant differences revealed across time (*p* > 0.05) and no significant condition × time interactions (*p* > 0.05) for any variable.

When comparing the relationships between the CMJ AS and CMJ NAS within each variable, during each test session, there was a very large significant positive correlation of RSI_Mod_^FT^ (Test Session 1: r = 0.803, *p* < 0.001; Test Session 2: r = 0.783, *p* < 0.001) and a very large significant positive correlation of RSI_Mod_^IMP^ (Test Session 1: r = 0.789, *p* < 0.001; Test Session 2: r = 0.722, *p* < 0.001), while there was a significantly large positive correlation of FT:CT (Test Session 1: r = 0.669, *p* < 0.001; Test Session 2: r = 0.621, *p* < 0.001).

There was a significant positive correlation between RSI_Mod_^FT^ and RSI_Mod_^IMP^ during both the CMJ AS (Test Session 1: r = 0.878, *p* < 0.001; Test Session 2: r = 0.925, *p* < 0.001) and CMJ NAS (Test Session 1: r = 0.986, *p* < 0.001; Test Session 2: r = 0.980, *p* < 0.001). There was a significant positive correlation between RSI_Mod_^FT^ and FT:CT during both the CMJ AS (Test Session 1: r = 0.958, *p* < 0.001; Test Session 2: r = 0.951, *p* < 0.001) and CMJ NAS (Test Session 1: r = 0.969, *p* < 0.001; Test Session 2: r = 0.965, *p* < 0.001). There was also a significant positive correlation between RSI_Mod_^IMP^ and FT:CT during both the CMJ AS (Test Session 1: r = 0.864, *p* < 0.001; Test Session 2: r = 0.910, *p* < 0.001) and CMJ NAS (Test Session 1: r = 0.961, *p* < 0.001; Test Session 2: r = 0.960, *p* < 0.001).

**Figure 1:**
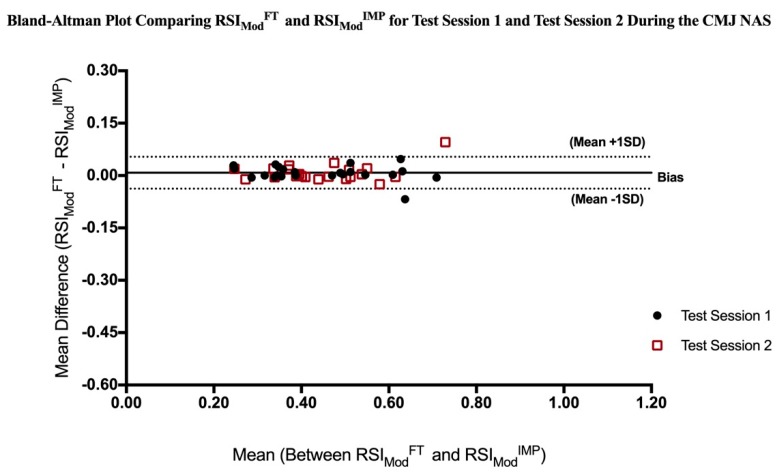
Bland-Altman Plot comparing RSI_Mod_^FT^ and RSI_Mod_^IMP^ during the CMJ AS. CMJ = Countermovement Jump; AS = arm swing; RSI_Mod_^FT^ = Reactive Strength Index Modified, computed via the flight time method; RSI_Mod_^IMP^ = Reactive Strength Index Modified, computed via the impulse-momentum method; SD = Standard deviation.

There were no significant differences between RSI_Mod_^FT^ and RSI_Mod_^IMP^ during either the CMJ NAS or the CMJ AS (*p* > 0.05). The Bland-Altman Plot in Figure 1 outlines an average measurement bias of 0.008 ± 0.02 during the CMJ NAS. Measurements of RSI_Mod_ by the flight time method (RSI_Mod_^FT^) ranged between −0.038 less and 0.054 greater than measurement by the impulse-momentum method (RSI_Mod_^IMP^) for 95% of individuals assessed during the CMJ NAS.

**Figure 2:**
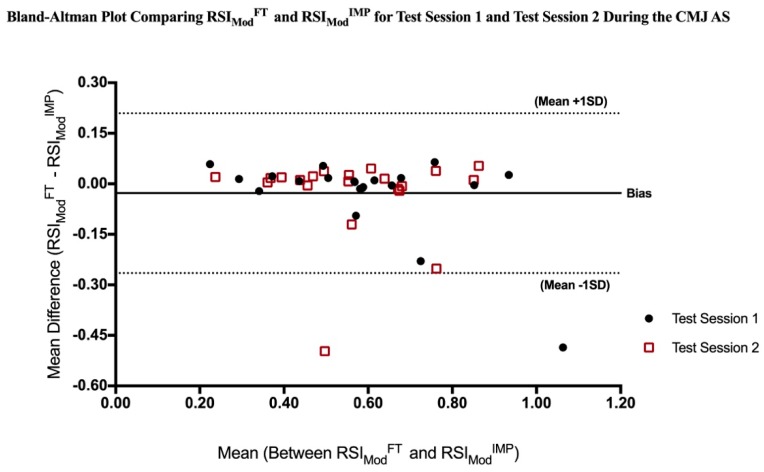
Bland-Altman Plot comparing RSI_Mod_^FT^ and RSI_Mod_^IMP^ during the CMJ AS. CMJ = Countermovement Jump; AS = arm swing; RSI_Mod_^FT^ = Reactive Strength Index Modified, computed via the flight time method; RSI_Mod_^IMP^ = Reactive Strength Index Modified, computed via the impulse-momentum method; SD = Standard deviation.

As illuminated by the Bland-Altman Plot in Figure 2, the average measure bias between RSI_Mod_^FT^ and RSI_Mod_^IMP^ during the CMJ AS was −0.028 ± 0.12. In addition, measurements of RSI_Mod_ by the flight time method (RSI_Mod_^FT^) varied between −0.265 less and 0.209 greater than measurement by the impulse-momentum method (RSI_Mod_^IMP^) for 95% of individuals assessed during the CMJ AS.

## 4. Discussion

The main findings of the present study were (a) a significant increase in RSI_Mod_^FT^, RSI_Mod_^IMP^, and FT:CT during the CMJ AS compared to the CMJ NAS; (b) a large correlation within RSI_Mod_^FT^, RSI_Mod_^IMP^, and FT:CT between jump protocols; (c) a large significant positive correlations among RSI_Mod_^FT^, RSI_Mod_^IMP^, and FT:CT, during both the CMJ AS and the CMJ NAS; and (d) RSI_Mod_^FT^ and RSI_Mod_^IMP^ demonstrated superior limits of agreement during the CMJ NAS compared to the CMJ AS.

The present study performed a novel comparison of RSI_Mod_^FT^, RSI_Mod_^IMP^, and FT:CT outcomes during the CMJ NAS compared to the CMJ AS. As hypothesized, the present study illuminated statistically significant increases, all of the medium effect sizes, in RSI_Mod_^FT^, RSI_Mod_^IMP^, and FT:CT during the CMJ AS compared to the CMJ NAS. Improvements in performance during the CMJ AS compared to the CMJ NAS is persistent throughout the literature [15,16,17,18,19], such as the increase in jump height of 10–12% [35]. Likewise, the velocity at take-off has been shown to be 6–10% greater during the CMJ AS [16,18,36]. Similarly to prior literature, the present investigation observed increases of 20%, 24%, and 11%, in RSI_Mod_^FT^, RSI_Mod_^IMP^, and FT:CT, respectively during the CMJ AS. While a variety of theories have been postulated as responsible for the improvements in performance during the CMJ AS, enhancements are likely the result of several mechanisms operating simultaneously. Early work by Payne [37] proposed the ‘transmission of force’ theory in which the upward acceleration of the arm swing increases reciprocal downward forces exerted through the body, increasing ground reaction forces and ultimately leading to a greater vertical velocity of the center of mass. While intuitive, this theory is likely an oversimplification, as newer work by Lees et al. [18] offers a more complex explanation, involving a series of events that allows the arms to build energy early in the jump and transfer that energy to the rest of the body in the later stages of the jump. Alternative theories include the eccentric stretching phase perhaps leading to a potentiation effect, with an increase in myoeletric activity during the subsequent concentric contraction [16], while others have speculated the significant increase in tension during the onset of the concentric contraction may result in enhanced chemical energy availability for force generation [35]. Regardless of the mechanisms at play, the observed increase in performance during the CMJ AS in the present study offers novel insight into maximal capacities that may be more directly related to performance during sport, beneficial to applied practitioners and researcher. 

Importantly, the present study identified only a large correlation, suggesting only about 38–64% of the shared variance between the CMJ protocols. The increase in the performance variables of RSI_Mod_^FT^, RSI_Mod_^IMP^, and FT:CT during the CMJ AS compared to the CMJ NAS, while lacking very large, or nearly perfect, correlations between jump performances indicates different information may be captured from the CMJ AS force-time signature not acquired from the CMJ NAS. Although much of the previous literature has used the CMJ NAS protocol to assess RSI_Mod_^FT^, RSI_Mod_^IMP^, and FT:CT [5,26,38], the present data suggests the inclusion of the CMJ AS protocol may identify alterations in maximal performance capacities. In addition, the CMJ AS may provide information independent from that obtained during the CMJ NAS and may relate more closely to the athlete’s expression of performance capabilities during the actual sporting event tasks, especially in sports incorporating a large vertical component. Furthermore, recognizing differences in performance between the CMJ NAS and CMJ AS will be essential in developing and comparing reference RSI_Mod_ and FT:CT values among various athletic populations [26].

Ultimately, these data suggest it may be necessary for practitioners to perform both CMJ protocols when assessing an athletes’ physical capacities. For example, practitioners may employ the CMJ NAS to evaluate acute changes in neuromuscular readiness, as previous literature has established less error of measurement. The CMJ AS may be more useful in to quantitating long-term changes in performance, such as changes in performance after a training program or alterations in performance between training phase, allowing coaches and practitioners to identify the changes in maximal performance capacities and where differences in performance are likely to be greater than acute changes.

The present study illuminated significant and strong positive relationships among RSI_Mod_^FT^, RSI_Mod_^IMP^, and FT:CT during the CMJ NAS, but uniquely identified a similar association among the variables during the CMJ AS. The findings of the present study support recent work by McMahon et al. [20] that also observed nearly identical Pearson’s correlation coefficients between RSI_Mod_^FT^ and RSI_Mod_^IMP^ (r = 0.980, *p* < 0.001), between RSI_Mod_^FT^ and FT:CT (r = 0.947, *p* < 0.001), as well as between RSI_Mod_^IMP^ and FT:CT (r = 0.944, *p* < 0.001) during the CMJ NAS. Moreover, these shared findings further endorse a significant and almost perfect positive relationship among RSI_Mod_ and FT:CT regardless of the computation method. Similarly, the relationship between RSI_Mod_^FT^ and FT:CT during the CMJ AS paralleled the results of the CMJ NAS, in an almost perfect fashion, which was expected considering the extensive use of congruent parameters to compute the variable. In contrast, the relationship between RSI_Mod_^FT^ and RSI_Mod_^IMP^, as well as between RSI_Mod_^IMP^ and FT:CT during the CMJ AS were still positively correlated and very large, however their relationship did not parallel to the same degree as the aforementioned relationships. The small relational disparities observed, especially during Test Session 1, coincide with observations of less reliability when using the impulse-momentum method to calculate jump height during the CMJ AS [10], however the variables remain strongly related. 

The present study found no significant differences between RSI_Mod_^FT^ and RSI_Mod_^IMP^ during the CMJ NAS or the CMJ AS. Important to note, these findings are in contrast to the recent findings by McMahon et al. [20] which observed a significant difference between variables using the CMJ NAS protocol, but only a trivial effect (*d* = 0.14). No differences between RSI_Mod_^FT^ and RSI_Mod_^IMP^ alone would indicate that practitioners could select either variable for player assessment. Interestingly, the CMJ NAS produced superior limits of agreement between RSI_Mod_^FT^ and RSI_Mod_^IMP^ compared to the CMJ AS, as visualized by the Bland-Altman Plots in Figure 1 and Figure 2. The CMJ NAS demonstrated a smaller average measurement bias and reduction in measurement difference variability. Differences in the limits of agreement are likely due to exaggerated variability in the velocity at take-off during the CMJ AS with the movement occurring at a greater velocity, leading to alterations in the reliability of the impulse-momentum computation of jump height [10]. Previous literature has identified the consequences of inaccurately pinpointing the instant of take-off by as little as 2–3 ms, which can manipulate an increase in variability of both velocity and displacement by as much as 2% [22,39]. The flight time method assumes center of mass is the same during take-off as landing, therefore differences in center of mass during take-off and landing can lead to an over estimation of the jump height calculation. Its speculated that the flight time method overestimates jump height, as the jumper’s center over mass is often higher at take-off than landing [29]. Previous literature has shown the center of mass is located at a higher relative position at take-off during the CMJ AS compared to the CMJ NAS [17], which may exacerbate differences in calculations in that the difference in center of mass during take-off are not paralleled upon landing. 

In addition, it is likely that the predetermined force thresholds used by the commercially available software influence the discrepancies between RSI_Mod_^FT^ and RSI_Mod_^IMP^, however collecting data at higher sampling frequencies, such as 1000 Hz in the present study is thought to reduce measurement error [22]. It should be mentioned that, it is also possible participants performed minute increases in hip, knee, or ankle flexion once airborne, not visible to the eye in real-time during the CMJ AS and which then did not occur during the CMJ NAS, resulting in a decreased association between variables within jump protocols [29,31]. However, the research team ruled jumps invalid when such characteristics were visible. In accordance with previous literature [10], these findings suggest the use of the CMJ NAS may be the more suitable protocol for athlete assessments when RSI_Mod_ is a key variable of interest, especially when evaluating acute day-to-day changes in neuromuscular functional performance. In addition, the enhanced limits of agreement during the CMJ NAS suggests the chosen computational method of RSI_Mod_ (RSI_Mod_^FT^ or RSI_Mod_^IMP^) when utilizing the CMJ NAS will likely exert a negligible influence on the RSI_Mod_ values.

Important to note, the present study found no differences between Test Session 1 and Test Session 2 and no significant condition by time interactions. These findings further support the inter-session reliability of the variables in the present study, as reported elsewhere [10].

The present study is not without limitations. This investigation examined a relatively small, homogenous sample of skilled jumpers. In addition, the levels of agreement among variables may be influenced by the software used for analysis. Finally, training loads were not quantitively confirmed with an athlete monitoring technique, such as internal or external training load measures [6].

In conclusion, the present study offers several key findings useful to applied practitioners using the CMJ with increasingly available commercial force platform technology to evaluate changes in performance and fatigue. The increases in RSI_Mod_^FT^, RSI_Mod_^IMP^, and FT:CT evident during the CMJ AS illuminate the potential use of the CMJ AS to reveal changes in maximal performance that may translate more closely to sport specific tasks, such as assessing performance changes after a training block or between training phases. In addition, the observed relationship within RSI_Mod_^FT^, RSI_Mod_^IMP^, and FT:CT between the CMJ AS compared to the CMJ NAS suggests each jump protocol may provide novel insight valuable to assessing an athlete’s physical capacities. In addition, the present investigation identified the strong relationship among RSI_Mod_^FT^, RSI_Mod_^IMP^, and FT:CT during both the CMJ NAS and CMJ AS. Furthermore, it appears the RSI_Mod_^FT^ and RSI_Mod_^IMP^ exhibit greater levels of agreement during the CMJ NAS compared to the CMJ AS. Practically, these findings indicate that either RSI_Mod_ or FT:CT may be utilized to monitor changes in neuromuscular function and performance, but it is unnecessary to include both, as they may provide similar information about an athlete’s force-time characteristics, conceivably making their simultaneous inclusion during a player assessment redundant.

## Figures and Tables

**Table 1:** Results for the RSI_Mod_^FT^, RSI_Mod_^IMP^, and FT:CT during both the CMJ AS and CMJ NAS.

Variable	CMJ AS		CMJ NAS	
	Test Session 1	Test Session 2	Test Session 1	Test Session 2
RSI_Mod_^FT^	0.577 ± 0.20	0.567 ± 0.17	0.458 ± 0.13	0.451 ± 0.12
RSI_Mod_^IMP^	0.607 ± 0.25	0.573 ± 0.18	0.451 ± 0.14	0.442 ± 0.12
FT:CT	0.773 ± 0.19	0.766 ± 0.19	0.672 ± 0.13	0.667 ± 0.13

CMJ = Countermovement Jump; AS = arm swing; NAS = no arm swing; RSI_Mod_^FT^ = Reactive Strength Index Modified, computed via the flight time method; RSI_Mod_^IMP^ = Reactive Strength Index Modified, computed via the impulse-momentum method; all data is reported as mean ± standard deviation.
